# Financial Independence and Academic Achievement: Are There Key Factors of Transition to Adulthood for Young Higher Education Students in Colombia?

**DOI:** 10.3389/fpsyg.2020.01330

**Published:** 2020-06-30

**Authors:** Mónica-Patricia Borjas, Carmen Ricardo, Elsa Lucia Escalante-Barrios, Jorge Valencia, Jose Aparicio

**Affiliations:** Department of Education, Universidad del Norte, Barranquilla, Colombia

**Keywords:** psychological autonomy, self-determination, human agency, academic achievement, transition to adulthood, financial independency

## Abstract

Autonomy is conceptualized as the need for agency, self-actualization, and independence. Nowadays, financial independence and academic achievement for young populations may be considered as key aspects in the transition to adulthood in response to some contextual demands of different cultural environments. By means of a multi-level model, the present study aims to determine the influence and contribution of factors at individual level (e.g., sex, age, socioeconomic status, family financial support, awarded scholarships, personal finance, student loans) and school level (e.g., program quality, online programs, face to face programs) on the academic achievement of young higher education Colombian students. Data come from the scores of the national standardized academic achievement test administered in 2018 in Colombia. The sample included 234,386 students enrolled in 3,389 higher education institutions in Colombia. After controlling the effects of program quality, and the student’s previous academic abilities and socio-economic conditions, results showed that students with scholarships had higher scores than financially dependent students (those who had student loans) and financially independent students (those who self-funded their studies or who worked during the week) who had low scores in the national standardized academic achievement test.

## Introduction

Moving on into adulthood implies to learn building a personal life project facing, progressively, the need for an increasing psychological and social independence. It requires learning to recognize the personal aims and the moral criteria and standards we will follow to achieve those aims, balancing the satisfaction of own needs against other people’s needs and aims (Chirkov, 2011).

This process of moving from a childhood dependence status to one of adulthood independence is especially linked to the autonomy development understood as the capability of self-determination (Ryan and Deci, 2002), personal agency (Martin et al., 2010), and self-sufficiency (Shim et al., 2010).

Although there are cultural differences that may indicate different ways and times, the entrance into the labor market is generally used as a reference for the transition to autonomy for youth (Bea and Yi, 2018). However, social changes have gradually enabled young people to access to university education and, as a consequence, the economic dependence has been prolonged, in such a way that some authors have proposed an intermediate developmental stage named emergent adulthood (Arnett, 2000).

But what happened to youngsters who must combine their university studies with work against those who continue being economically dependent during this stage? Will living this experience accelerate their autonomy, positively affecting variables such as academic performance, when assuming their studies with increased responsibility? Is it necessary and desirable to prolong adolescence dependence in emergent adulthood (Carlson, 2014) in terms of making optimal use of the university experience?

For that reason, it is important to carry out research that may explain the possible impact of financial independence and academic success in university students. Some researchers have examined this association (Canton and Blom, 2010; Melguizo et al., 2016). However, existing literature about this topic is limited (Canton and Blom, 2010).

### Academic Performance and Autonomy: School and Students Factors

According to Yu and Levesque-Bristol (2018) and Yu and Levesque-Bristol (2020), autonomy has a greater impact on academic performance than other personal factors. Thus, its development has become one of the main aims of all education levels (Zimmerman, 2002; Toro, 2004).

There are educational scenarios that contribute to develop autonomy because they offer the students decision-making possibilities. Yu et al. (2018) state that students of social sciences and humanities tend to be more self-determined and autonomous when comparing to students of careers related to business. Thus, they highlight the importance of promoting more humanistic learning environments in certain academic disciplines.

Concerning the modality of academic programs, in all of them, students need regulate their learning process, but some research has found that educational environments offering high flexibility degrees, as it occurs in virtual programs, provide greater opportunities to make decisions independently (Sauerwein, 2017; Yuan and Kim, 2018; Bonem et al., 2019), and make learners assume regulatory behaviors to obtain an impact on their achievement and performance (Cazan, 2014; Roddy et al., 2017). This is reinforced by Mostrom and Blumberg (2012) and Ryan and Deci (2017), who claim that self-regulatory learning behavior is important in off-campus programs and is associated with higher academic performance when it is compared to more controlled environments, as face-to-face programs. However, in virtual and distance-learning modalities, it is necessary to have greater autonomy and responsibility for achieving learning objectives when assuming regulatory behaviors. On the other hand, according to Dziuban and Moskal (2011a; 2011b), what has been demonstrated is that modality is not an effective predictor of academic success, and that the stronger predictor is the previous academic performance (Dziuban and Moskal, 2011a; Xiao, 2018; Paul and Jefferson, 2019; Torres and Parra, 2019).

According to Kirmizi (2013), in traditional distance-learning education, autonomy acquires more importance because students manage and lead their learning process; they are alone and far from their classmates and tutors, and without the technological mediation that facilitates the permanent interaction among the actors of the process, it could be favored learning process desertion. Thus, learners must have the necessary abilities to lead their learning process (Roddy et al., 2017). Gottardi (2015) considers that autonomy in distance education is developed during all the formation. In the research by Leaño and Jaramillo (2018), it could be appreciated that some students, in the most advanced levels, in this modality show typical abilities of autonomy in their process of formation.

### Financial Independence as Key Element for the Development of Autonomy and Academic Performance for Emerging Adults

Some authors (McGoldrick et al., 2016; Watson and Barber, 2017) claim that becoming a fully functional autonomous adult requires the criterion of being economically independent. In this regard, grant aids may be considered as a key factor for promoting equality, diversity and financial well-being for emerging adults (Alon, 2007). Alon (2005, 2007) developed a conceptual framework to assess the impact of financial aid on academic outcomes taking into consideration the blending effect of aid eligibility and types of aid (i.e., grants, loans, work-study) after controlling key students’ characteristics (i.e., race/ethnicity, sex, SAT scores, college grade-point average, need-based financial aid status, and date of graduation, parental education, and others). In terms of grants, the author highlights that it is important to distinguish between need-based vs. merit-based aid because of the individual characteristics of the recipients (Alon, 2005). Individual characteristics like race, ethnicity, and disadvantage in economic status may affect negatively academic outcomes (i.e., retention, graduation rates), especially for minority students like African-Americans and Hispanics living in disadvantaged conditions in the U.S. who tend to be more sensitive to the accessibility to grant aids (Alon, 2007).

A recent systematic review by Nguyen et al. (2019) concluded that grant financial aid has a positive effect on a number of postsecondary academic outcomes such as persistence and degree completion. Zhu et al. (2019) recognize these positive effects in the academic outcomes of University students from Texas, beneficiaries of the SCOPE grant program for STEM programs.

Regarding loans, Canton and Blom (2010) reported that Mexican college students who have access to financial aid, package (loans and scholarships) through the program “Society for the Promotion of Higher Education” (SOFES, for its acronym in Spanish), funded by the World Bank, increased the grade point average by 0.17 on a 10 point-scale, which is a 3% improvement. SOFES recipients reported higher academic performance than students without credit from SOFES. Melguizo et al. (2016) examined the impact of the program “Access with Quality to Higher Education” (ACCESS for its acronym in Spanish) on academic outcomes in low-income college students in Colombia (i.e., enrollment rates, percentage of courses passed, dropout rates). Findings showed that access to ACCESS, which is the national-level subsidized loan program, increased the percentage of courses passed by the students at the margin by 3% points approximately. Overall, the ACCESS recipient increased by 4% points the proportion of coursed passed. In the U.S., Denning (2017) examined the effect of financial aid for financially independent students on their graduation and college enrollment. Using a sample of students enrolled in US public universities, the author found that the students who receive financial aid are more likely to graduate in the year they turn 24 than those who do not receive loans. Finally, a recent study carried out by Graziosi et al. (2020) with a sample of Italian university students also showed a positive effect of receiving grant financial aid on the likelihood of graduating, achieving a higher number of credits, and avoiding dropout.

On the other hand, Bennett et al. (2015) reported that students in the U.S. who received loans may be worried about paying for their college debts. In fact, these groups of students showed 4.5% points lower in economic course grades than their counterparts. Fuse (2018) examined the effect of university debts on academic performance, which is low especially for students of low socioeconomic status. Besides, a greater amount of owned debts seems to be related to lower academic performance (Alon, 2007) in college students and greater distress (Shim et al., 2009; Fuse, 2018), Overall, these findings showed that financial stress negatively affected college students’ academic performance (e.g., Rafidah et al., 2009; Bennett et al., 2015), especially minorities, women, and first generation of immigrants who have shown higher levels of financial stress and lower scores (Bennett et al., 2015).

When the family subsidizes the university studies, the student has the option of studying in his/her place of origin or going to another place. Family support is an important factor that can provide socioemotional support and economic support in college (Cheng et al., 2012; Cui et al., 2019); however, this postpones the financial independence of the emerging adult. Cheng et al. (2012) reported that college students’ cumulative GPA scores were not predicted by the perceptions of family economic support; however, the interaction of family economic support and family support is a significant predictor of students’ grade point average. Hamilton (2013) explained a negative relationship between family economic support and grades in a national representative sample. The author reported that when parental economic support increased, a student’s grade point average decreases (*b* = −2.233, *p* < 0.001), especially women and older students from Hispanic nations who significantly showed lower grade point averages than their counterparts. This is contrary to the findings by Dahl and Lochner (2012) and Cui et al. (2019) that state that young adults from rich families have a better academic performance. On the other hand, Roksa and Kinsley (2019) reported that there was no significant association between family financial support and grade point average in a sample of low-income college students in the U.S. Overall, there is a lack of inconsistent evidence about financial-economic support in college students (Cheng et al., 2012; Hamilton, 2013).

University students who migrate with financial family support show a significant tension between autonomy, considered from the self-sufficiency, and dependence, strengthening confidence and determination (Gamallo and Nuñez, 2013; Torcomian, 2016; Rangel et al., 2019). However, migration may generate negative effects on academic performance (Edwards and Baker, 2014; Muñoz Montes and Marín Catalán, 2018).

Another way of achieving financial independence is the tying of emergent adults to the labor market. In Western societies, the economic weight of financing university studies is supported by family rather than the State (Darmody and Smyth, 2008), and in poor countries, as the majority of Latin American countries and Colombia is not the exception, this economic weight is displaced to the student. The literature shows that the phenomenon of financial independence is more marked on young people coming from the poorest and more vulnerable sectors (Dovey et al., 2017). When the family cannot support the cost of university education, it is the student who assumes this cost. As it is claimed by Fuse (2018), university young people who work part-time or full-time, especially in Western societies, do it because of financial need. This situation makes them become financially independent and/or financially responsible for others; as Hardin (2008) states, this is identified as a risk factor for the academic performance of university students.

Results about the effects of working on university students are contradictory (Padgett and Grady, 2009). Carrillo and Ríos (2013) state that there is no difference between students who work and those who do not. In the same way, Arano and Parker (2008) claim that the difference will only happen if the number of hours is above the threshold. About this topic, Greenberger and Steinberg (1986) and Barling et al. (1995) place the threshold that balances the study, work, and other life activities, in 20 h, while Choy (2002) place it about 15–25 h per week. Beyond this threshold, negative effects may occur (Curtis and Williams, 2002). One of these affects the academic performance (Chinyakata et al., 2019; Vicencio and Banaag, 2019). Bradley (2006) refers to the affectation to students’ physical, mental and emotional health caused by an increasing number of hours devoted to work.

About the effect on academic performance, Wenz and Yu (2010) state that this impact will depend on the reason the students have to decide to work. Wang et al. (2010) and Carrillo and Ríos (2013) assert that such an impact may be conditioned by other factors such as the quality or the kind of work carried out. About the former, Bradley states that the satisfactory quality of work generates positive effects on students’ results. About the latter, Bradley (2006) and Cheng and Alcantara (2007) link these effects to the proximity or association of the type of work to the university career. In general, Dundes and Marx (2006) recognize the positive effects of work in university students. Fazio (2004) points out at the work positive incidence on the students when the working time is moderate, especially if the work is associated with a professional career.

Among the advantages the work may generate is recognizing the self-confidence (Bradley, 2006), specifically the leadership (Salisbury et al., 2012). At the same time, the leadership is associated to the autonomy, which improves the general academic performance (Deng et al., 2020). Salisbury et al. (2012) state that the work may generate positive effects on autonomy development. They mention the benefits for the development of confidence and self-sufficiency when working during the studies. However, they assert that excessive work may limit students’ participation in co-curricular activities and peer interaction.

Finally, the achievement of financial independence for college students may be determined by socio-cultural and/or economic conditions (Blakemore and Mills, 2014; Carlson, 2014; Vera, 2014; Apeltauer and Senyildiz, 2015; Arnett, 2015; Fuse, 2018), which affect academic performance (Alon, 2007; Canton and Blom, 2010).

According to the context described, the study aims to determine the influence and contribution of factors at individual level (e.g., sex, age, socioeconomic status, family financial support, awarded scholarships, personal finance, student loans, students who prepared themselves for national tests) and school level (e.g., online programs, face-to-face programs) on the academic achievement of young higher education Colombian students.

## Materials and Methods

### Design and Measure

The study used a quantitative research approach with a correlational scope. It aims at discriminating adequately the effect of financial autonomy on Colombian university students’ learning result. Characteristics of both subjects and academic programs were jointly analyzed because these two units of analysis belong to different levels of aggregation. Variables of 139,143 students who took the *ICFES SABER PRO* (Instituto Colombiano para la Evaluación de la Educación – ICFES, 2019) test in 2018 were analyzed. Complete information about them was available, as well as the possibility of having access to their academic background when they took the test *SABER 11* applied at the end of secondary school, about 2006–2014 (Level 1). This population was distributed in 3,389 university programs registered in the Information National System of Higher education (Level 2).

Following the Colombian legal norms, the State Examination for higher education, SABER PRO, is mandatory to obtain the title at the pre-graduate level. This text is oriented at proving the development degree of generic and specific competencies of students who have approved at least 75% of the academic credits of the program they are studying (Instituto Colombiano para la Evaluación de la Educación – ICFES, 2019).

According to this and considering that the use of linear regressions in hierarchical structures of variables may be inadequate^1^ when the aim is to analyze two analysis units in conjunction (Murillo Torrecilla, 2008), in this study, multilevel or linear hierarchical models were used. These models enable us to address variables in diverse levels of aggregation simultaneously, and they are well-recognized for their usefulness when the units of analysis are organized in hierarchical structures (Grilli and Rampichini, 2009; Mertens, 2014; Jongbloed and Lepori, 2015), as the case of academic institutions and programs. Essentially, multilevel models are a set of classical linear models for each level, where models of both levels are related in such a way that the coefficient of the first one is incorporated in the second level as explanatory variables.

### Procedure

This study aims at determining if the performance in the assessed competencies in the State Examination SABER PRO is related to students’ financial autonomy, once other relevant characteristics regarding the social and educational context, and conditions of the program in which they have studied, are considered. The information about these variables was obtained from the databases of the Colombian Institute for Evaluation of Education (ICFES, by its acronym in Spanish) collected during the application of State Examinations at the end of the secondary school (SABER 11) and the end of the professional training (SABER PRO). The variables used in the study are as follows:

Level 1-variables (students)

• SPRO: Average score of generic competencies assessed in SABER PRO: critical reading, quantitative reasoning, writing, citizen competencies, and English.
• INSE: Students’ socio-economic level calculated from information about holding goods and services, collected in the inscription forms
• S11: Index of Saber 11 results in the areas of the common nucleus, Language, Mathematics, Social Sciences, Natural Sciences, Philosophy, English
• AGE: Student age in years.
• SCH: The student finances their studies by a scholarship.
• LAN: The student finances their studies by a loan.
• OWN: The student finances their studies by own resources or income.
• WRK: Student’s working time in the week.
• HOM: The student lives in a temporary home.
• HEA: The student is financially responsible for the home in which they live.
• CIT: The student lives in the city in which the program is offered.

Level 2-variables (Program)

• ACC: Certification of High Quality by the National Minister of Education
• MOD: Program modality or methodology (face-to-face, distance, or virtual education)

Because it is not possible to have a direct measure of the financial autonomy, the variables AGE, SCH, LAN, WRK, HOM, HEA, and CIT will be assumed as proxies of this phenomenon, while the variables INSE, S11, ACC, and MOD will enable us to control the important effects on the score that may derive from students and program characteristics.

Level 1 model gives an account of the estimation of SABER PRO global score of the student i in the program j considering their characteristics (x), their previous academic abilities (Saber 11), their socio-economic level, and the variables referent to financial autonomy. To predict students’ overall Saber Pro scores, we were based on a vector of covariates that include student-related control variables (level 1): prior cognitive outcomes (S11), socioeconomic conditions (INSE), and program conditions (level 2): certification in quality (ACC) and methodology (MOD). By controlling the effect of these covariates, the incidence of the variables of interest, related to economic independence, in this case AGE, SCH, LAN, OWN, WRK, HOM, HEA, and CIT, will be estimated.

Data organization, as well as the descriptive analysis and the estimation of the models, was carried out by using the statistical packages Stata 15 and SPSS 25.

## Results

This section has been divided into two parts. Firstly, it shows the general behavior of the variables of interest related to the financial autonomy found in SABER PRO database. Secondly, it shows the results of multilevel model estimates, which report about the effect of some characteristics related to autonomy on the result of the measure of competencies at the end of the professional training.

### Variables Related to Students’ Financial Autonomy

Table 1 shows the distribution of assessed students according to each variable HOM, HEA, CIT, SCHO, LOAN, and OWN, as well as the average score for each case.

**TABLE 1:** Profile of students’ variables: HOM, HEA, CIT, SCHO, LOAN, and OWN.

**Variable**	**Group**	**n**	**%**	**Mean SPRO**	**Mann-Whitney test asymp. sig. (2-tailed)**
HOM	No	19,629	18.95	149.30	0.000
	Yes	83,934	81.05	150.84	
HEA	No	93,778	90.55	151.64	0.000
	Yes	9,785	9.45	140.10	
CIT	No	31,916	30.82	145.21	0.000
	Yes	71,647	69.18	152.92	
SCHO	No	86,153	83.44	149.96	0.000
	Yes	17,101	16.56	153.56	
LOAN	No	69,144	66.96	151.70	0.000
	Yes	34,112	33.04	148.23	
OWN	No	66,022	63.94	153.56	0.000
	Yes	37,234	36.06	145.22	

**Source: Authors based on the data of Instituto Colombiano para la Evaluación de la Educación – ICFES (2019).**

It can be observed that the students who do not live in the city in which the program is offered, who live in a temporary home, and those who are financially responsible for their family or home, had lower average scores (SPRO). Regarding financing, scholarship students represent 16.6% of the population; these students achieve an average score significantly higher than the rest of the population. On the other hand, students who resort to other sources to finance their studies, such as loans or own resources, have lower scores, even though considering these financing alternatives are not mutually exclusive. Besides, certain trends regarding students’ age (AGE), and the working time during the week (WORK) can be identified, both variables being key in the transition from higher education to the productive life. Concerning age, a negative correlation with the global score (*r* = −0.268, *p* = 0.000) is observed, with significant decreases in the global score from 24 years (see Table 2).

**TABLE 2:** Age range and SPRO average.

**Age (years)**	**Mean**	**N**	**Std. deviation**
<=19	160.33	54	25.407
20–21	156.85	18,490	22.661
22–24	155.59	34,137	24.005
25–26	146.97	36,201	22.370
27–28	141.39	11,612	21.278
29+	133.06	3,069	21.490

**N* = *103,563; F = 1527,275; p-value = 0.000. Eta = 0.262; eta Squared = 0.069. Source: Authors.**

Regarding working hours, two-thirds of students say they work full-time or part-time. About 40% of them worked 30 or more hours per week. As for the academic result, a negative trend is observed, with significant differences between those who do not work and those who do it. It is noteworthy that the differences among the scores tend to decrease between those working full-time and part-time (Table 3).

**TABLE 3:** Working hours per week and SPRO average.

**Hours worked per week**	**Mean**	**N**	**Std. deviation**
Not work	156.07	23,947	24.843
<10	150.11	12,909	25.409
11–20	149.32	18,814	23.805
21–30	148.41	15,608	22.828
+30	148.37	32,285	21.611

**N = 103,563; F = 447.422; p-value = 0.000. Eta = 0.130; eta Squared = 0.017. Source: Authors.**

### Estimate of Effects of Financial Autonomy on SABER PRO Results

Below are the results of the adjusted model that includes the variables of level 1 and 2, where the students’ characteristics (INSE, SB11) are controlled. Regarding this, it is observed that the effect of students’ previous academic antecedents is the variable that most affects the result: in the same way, their socio-economic conditions have a negative effect on Saber pro average. In summary, higher scores in Saber 11 (previous academic antecedent for entrance to university), as well as better socio-economic conditions, are related to higher scores in the test applied at the end of the professional training.

Regarding the characteristics of the programs, it is observed that better quality conditions (ACC) evaluated from the perspective of the country’s quality assurance system lead to higher average scores in the test. As regards the type or modality, it is found that virtual programs have higher scores than traditional distance programs (between 2.45 and 4.3 points) and lower scores than face-to-face programs. However, the difference between virtual and face-to-face programs could not be conclusive considering the *p*-value and the confidence intervals link to the estimator of this variable (Table 4).

**TABLE 4:** Estimate of the model.

**Parameter**	**Estimate**	**Std. error**	**Standardized coefficients**	**df**	**t**	***p*-value**	**95% Confidence interval**	
							**Lower bound**	**Upper bound**
Intercept	178.965	0.857		12284.473	208.923	0.000	177.286	180.644
INSE	0.183	0.006	0.008	101075.116	30.730	0.000	0.171	0.194
SB11	19.004	0.076	0.805	102755.959	248.995	0.000	18.855	19.154
ACC (Yes)	0.615	0.128	0.026	92445.597	4.788	0.000	0.363	0.867
MOD (Campus)	1.096	0.443	0.051	90417.070	2.476	0.013	0.228	1.963
MOD (Distance)	−3.367	0.467	−0.142	93135.065	−7.206	0.000	−4.283	−2.451
**MOD (Virtual)**								
AGE	−1.435	0.022	−0.060	103210.555	−64.283	0.000	−1.478	−1.391
HOM (Temporary)	−1.379	0.122	−0.057	103216.223	−11.301	0.000	−1.618	−1.140
HEA (Yes)	−0.953	0.168	−0.040	103088.883	−5.683	0.000	−1.282	−0.624
CIT (Different)	−0.709	0.115	−0.029	102981.272	−6.178	0.000	−0.934	−0.484
SCHO (yes)	1.164	0.130	0.049	103226.560	8.970	0.000	0.910	1.418
LOAN (yes)	−0.295	0.106	−0.013	103090.693	−2.781	0.005	−0.502	−0.087
OWN (yes)	−0.206	0.110	−0.007	103206.366	−1.879	0.060	−0.421	0.009
WORK (yes)	−0.937	0.116	−0.038	103134.686	−8.072	0.000	−1.165	−0.710

**N = 103,342; ICC = 0.084.**

Once the effects of relevant characteristics of students and programs were controlled, each variable available in Saber Pro measurement related to financial autonomy was examined. Firstly, it was found the age (AGE): according to the model results, one year more of a student’s age causes a decrease in the average score close to 1.4 points. As mentioned earlier, the decrease in scores is more pronounced 24 years when the differences between the average scores are statistically significant. On the other hand, a negative effect on the average score of those students who live in temporal homes (HOM) and/or those who study in a place different to their place of residence (CIT) was observed.

As regards financing mechanisms, results show that once the most relevant contextual variables are controlled, students who finance their education through a scholarship achieve higher scores in SABER PRO test by about 1 and 1.5 points. By contrast, the study found a negative effect on the global score in those students who finance their studies by own resources (OWN). About loans, although a possible negative effect (LOAN) is observed, the associate *p*-value estimator of this variable is higher than 0.05. Similarly, evidence of a negative effect of full-time or part-time work (WORK) on the average score was found.

## Discussion

The current study examined the association between the scores of national standardized learning tests and a set of variables associated with the dependence-independence finance in college students, starting from the supposition that independence, which is characteristic of a complete adulthood (Arnett, 2000), would promote students’ greater autonomy that in turn would impact positively on their academic results.

However, in this research as in other previous ones (Bradley, 2006; Chinyakata et al., 2019; Vicencio and Banaag, 2019), findings show that those students with financial autonomy, who support their studies with own resources or working full-time or part-time, achieved lower scores in the State Examination than their granted peers (Hossler et al., 2009), regardless of the form of financial aid (need-based vs. merit-based aid).

Other studies have also found that students who have access to this type of financial aid increased their academic performance (Canton and Blom, 2010; Melguizo et al., 2016). For instance, Canton and Blom (2010) reported that Mexican college students who are SOFES (loans and scholarships for Mexican Higher of Education funded by the World Bank) recipients increased their grade point average more than students without a credit from SOFES. In the Colombian context, Melguizo et al. (2016) showed that ACCESS program recipients (national-level subsidized loan program) increased the percentage of courses passed at the margin by 3% points approximately. It is important to highlight that these research studies conducted in Latin countries did not measure academic performance using the scores of national standardized learning tests, which might suggest that loans could influence academic performance in different ways depending on the selected outcome to operationalize the construct. Additionally, the current study did not examine the association between academic performance and each type of loan program like private loans and/or national-level subsidized loans, which also could address different findings.

Of course, it cannot be stated that the great autonomy, both economical and personal, of students who work is a variable that negatively affects the scores achieved in the examination at the end of their careers. It is likely to hypothesize that the influence weight of this variable is lower than the others (e.g., time devoted to study) that at the end act against their academic results (Rafidah et al., 2009; Bennett et al., 2015). For example, Bennett et al. (2015) reported that students in the U.S. who received loans may be worried about paying for their college debts. Unfortunately, the perception of financial worries is not a variable included in the national database; for this reason, it was not examined in the current model.

It seems that although the early entrance into the labor world could promote autonomy, it should come at its right time because to mix the academy with the work may fall away their possible positive effects. On the other hand, prolonging the dependency may have undesirable social consequences, for example, deficiency to act as a responsible citizen who makes social and political decisions independently. To balance the pros and cons, this greater transition time could and should be exploited as a crucial period for exercising the co-autonomy that eases the step from the dependence to the total autonomy.

Findings seem to be consistent with the hypothesis that the extent of studies until university level has conditioned the emerging of the intermediate stage of emergent adulthood (McGoldrick et al., 2016; Watson and Barber, 2017) that prolongs the adolescent dependency for the sake of achieving academic aims.

This hypothesis seems to be confirmed when analyzing another variable being that theoretically linked to a great autonomy; it was expected to have a positive effect on academic results: living outside the family nucleus while studying in the university. Nevertheless, as with economic independence, to study far from the family seems to negatively affect academic results.

It could be supposed that the absence of family emotional support could be the key factor causing this effect, but there are studies for Alnabhan et al. (2010) and against it (Hamilton, 2013). Muñoz Montes and Marín Catalán (2018) found that the students who migrate for studying have lower academic results when they are compared to those who do not do it. But Edwards and Baker (2014) argued that Caribbean students that go out of their home to go to study in the United States are characterized by levels of self-determination and maturity, which positively impact their academic performance. These results are similar to those of Aguiar (2017) and Rangel et al. (2019).

Gamallo and Nuñez (2013) have found that their sample of university students valued the positive impact of migrating on their growing and autonomy development, but they also recognized that there continues to be a high dependence due to the need for family support.

Our hypothesis is that again the negative effect may be more associated with the time factor than to other variables. Torcomian (2016) also concluded that studying away from home implies an abrupt step from dependence to self-management, which supposes moving from adolescence to emergent adulthood in activities that go beyond the customary ones.

Another result was that age is a variable that seems to affect academic results because students older than 24 years achieved lower scores than their younger counterparts. Given that it was not possible to carry out a more detailed analysis of this relation, we can only hypothesize possible explanations for this finding. In this age group, it is likely that a great percentage of students with academic backwardness have diverse difficulties that at the end negatively affect their academic results. Besides, at this age, it is more probable that additional responsibilities such as parents’ role appear and, at the time passing, the importance of test results starts to be relativized.

The autonomy commonly considered as a predictor variable of academic success in virtual programs against face-to-face ones (Mostrom and Blumberg, 2012; Cazan, 2014; Roddy et al., 2017; Ryan and Deci, 2017) is not overwhelming in this study. In our findings, there is no conclusive evidence that supports the hypothesis that face-to-face programs have better results than their virtual counterparts. There are no educative models that are better than others. Everything depends on the quality criteria associated with each modality, in such a way that results and high performance in students’ education in virtual modality may be associated with factors like motivation, self-efficiency, and persistence (Francis et al., 2019).

As a relevant element, findings suggest that the context variables with greater weight in the result are university students’ academic antecedents as Dziuban and Moskal (2011a); Xiao (2018), Paul and Jefferson (2019), and Torres and Parra (2019) suggest, and the quality conditions of both institution and programs (Saavedra, 2009; Balapumi et al., 2016).

In general, there are at least two limitations to interpret results. The first one is related to the impossibility of stating that the increase of autonomy through self-management of different life aspects (studies, expenses, and so on) has a positive effect on the measure. The second limitation is that the test is not designed to collect directly these aspects – although it is defined as a competency test that informs, at least partially, about elements of know-how. However, it gives variables that allow recognizing valid patterns associated with its behavior in sound terms. Despite these limitations, the study is the first step to analyze certain types of autonomy concerning the results of the process of professional training in terms of learning. Future research might give light about the role of HEI about the strengthening of abilities, such as autonomy, which favor the healthy move to adulthood.

## Data Availability Statement

Publicly available datasets were analyzed in this study. This data can be found at the ICFES web page (www.icfes.gov.co and ftp.icfes.gov.co). It is important to highlight that original data have been modified and transformed in order to guarantee the confidentiality of unit of analysis. ICFES authorizes the use, transformation, and analysis of the information contained in its official web page provided that the source is quoted as ftp.icfes.gov.co. The contact is the Colombian Institute for Education Assessment – ICFES – solicitudesinformacion@icfes.gov.co (www.icfes.gov.co).

## Ethics Statement

Ethics approval was not required as data employed is extracted from a public repository. Similarly, according to numeral 7th of article 12 of Law 1324 of 2009, one of the functions of ICFES is to carry out research and studies about the assessment of education quality including both quantitative and qualitative aspects. In the same way, this regulation states that the great amount of data generated by these assessments constitutes a valuable input to carry out research about education quality that produces knowledge about relevant aspects of the education agenda and may help in the design of public policies and scholar practices. As a consequence, ICFES authorizes the use, transformation, and analysis of the information contained in its official web page provided that the source is quoted as ftp.icfes.gov.co.

## Author Contributions

M-PB led the manuscript writing process, theoretical framework construction, and participated in data analysis and discussion. CR co-conceptualized the research study, participated in data analysis, and discussion. EE-B led the methodological approach, participated in theoretical framework construction, data analysis, and contributed to the writing process. JV led the data analysis, co-led methodological approach, discussion, and contributed to the writing process. JA co-conceptualized the research study, co-led the theoretical framework construction, and contributed to the writing process, data analysis, and discussion. All authors contributed to the article and approved the submitted version.

## Conflict of Interest

The authors declare that the research was conducted in the absence of any commercial or financial relationships that could be construed as a potential conflict of interest.

## Footnotes

1 These models are supported on the assumption of independence of the observations, and as sharing the same context produces dependence of data which correspond to individuals, estimated standard errors of the traditional statistical test are underestimated, which will cause a significantly spurious result (Murillo Torrecilla, 2008).

## References

[B1] Aguiar D. A. (2017). *Estudiar Lejos de Casa: Guiìa Para Mejorar la Experiencia de ser Alumno Foraìneo.* México: ITESO.

[B2] Alnabhan M. Al-Zegoul E. Harwell M. (2010). Factors related to achievement levels of education students at Mu’tah University. *Assess. Eval. High. Educ.* 26 593–604. 10.1080/02602930120093913

[B3] Alon S. (2005). Model misspecification in assessing the impact of financial aid on academic outcomes. *Res. High. Educ.* 46 109–125. 10.1007/s11162-004-6291-x

[B4] Alon S. (2007). The influence of financial aid in leveling group differences in graduating from elite institutions. *Econ. Educ. Rev.* 26 296–311. 10.1016/j.econedurev.2006.01.003

[B5] Apeltauer E. Senyildiz A. (2015). Learner autonomy: Perceptions and attitudes of multilingual Turkish students training to become teachers. *Moder. Språk* 109 13–29.

[B6] Arano K. Parker C. (2008). How does employment affect academic performance among college students? *J. Econ.* 34 65–82.

[B7] Arnett J. J. (2000). Emerging adulthood. A theory of development from the late teens thorough the twenties. *Am. Psychol.* 55 469–480. 10.1037/0003-066X.55.5.469 10842426

[B8] Arnett J. J. (2015). *Emerging Adulthood: The Winding Road from the Late Teens Through The Twenties.* New York, NY: Oxford University Press.

[B9] Balapumi R. von Konsky B. R. Aitken A. McMeekin D. A. (2016). “Factors influencing university students’ self-regulation of learning: an exploratory study,” in *Association for Computing Machinery (ACM), Proceedings of the Australasian Computer Science Week Multiconference. Conference: the Australasian Computer Science Week Multiconference*, New York, NY, 10.1145/2843043.2843067

[B10] Barling J. Rogers K. Kelloway E. K. (1995). Some effects of teenagers’ part-time employment: The quantity and quality of work make the difference. *J. Organ. Behav.* 16 143–154. 10.1002/job.4030160205

[B11] Bea M. D. Yi Y. (2018). Leaving the Financial Nest: Connecting Young Adults’ Financial Independence to Financial Security. *J. Marriage Fam.* 81 397–414. 10.1111/jomf.12553

[B12] Bennett D. McCarty C. Carter S. (2015). The impact of financial stress on academic performance in college economics courses. *Acad. Educ. Leadersh. J.* 19 25–30.

[B13] Blakemore S. J. Mills K. L. (2014). Is adolescence a sensitive period for sociocultural processing? *Ann. Rev. Psychol.* 65 187–207. 10.1146/annurev-psych-010213-115202 24016274

[B14] Bonem E. M. Fedesco H. N. Zissimopoulos A. N. (2019). What you do is less important than how you do it: the effects of learning environment on student outcomes. *Learn. Environ. Res.* 23 27–44. 10.1007/s10984-019-09289-8

[B15] Bradley G. (2006). Work participation and academic performance: a test of alternative propositions. *J. Educ. Work* 19 481–501. 10.1080/13639080600988756

[B16] Canton E. Blom A. (2010). Student support and academic performance: experiences at private universities in Mexico. *Educ. Econ.* 18 49–65. 10.1080/09645290801931766

[B17] Carlson C. L. (2014). Seeking self-sufficiency: why emerging adult college students receive and implement parental advice. *Emerg. Adulth.* 2 257–269. 10.1177/2167696814551785

[B18] Carrillo S. Ríos J. G. (2013). Trabajo y rendimiento escolar de los estudiantes universitarios. El caso de la Universidad de Guadalajara, México. *Rev. Educ. Super.* 42 09–34.

[B19] Cazan A. M. (2014). “Self-regulated learning and academic achievement in the context of online learning environments,” *Paper presented at the 10th International Scientific Conference eLearning and Software for Education, Bucharest April 24-25*, Bucharest.

[B20] Cheng D. X. Alcantara L. (2007). Assessing working students’ college experiences: A grounded theory approach. *Assess. Eval. High. Educ.* 32 301–311. 10.1080/02602930600896639

[B21] Cheng W. Ickes W. Verhofstadt L. (2012). How is family support related to students’ GPA scores? A longitudinal study. *High. Educ.* 64 399–420. 10.1007/s10734-011-9501-4

[B22] Chinyakata R. Raselekoane N. R. Consolia R. R. (2019). Factors contributing to postgraduate students working and studying simultaneously and its effects on their academic performance. *Afr. Renaiss.* 16 65–81. 10.31920/2516-5305/2019/v16n1a4

[B23] Chirkov V. I. (2011). “Dialectical relationships among human autonomy, the brain, and culture,” in *Cross-Cultural Advancements in Positive Psychology: Vol. 1. Human Autonomy in Cross-Cultural Context: Perspectives on the Psychology of Agency, Freedom, and Well-Being*, eds Chirkov V. I. Ryan R. M. Sheldon K. M., (Dordrecht: Springer), 65–91. 10.1007/978-90-481-9667-8_4

[B24] Choy S. P. (2002). *Access & Persistence: Findings from 10 years of Longitudinal Research on Students.* Washington, DC: American Council on Education.

[B25] Cui X. Xiao J. J. Yi J. Porto N. Cai Y. (2019). Impact of family income in early life on the financial independence of young adults: Evidence from a matched panel data. *Int. J. Cons. Stud.* 43 514–527. 10.1111/ijcs.12536

[B26] Curtis S. Williams J. (2002). The reluctant workforce: undergraduates’ part-time employment. *Educ. Train.* 44 5–10. 10.1108/00400910210416192

[B27] Dahl G. Lochner L. (2012). The impact of family income on child achievement: evidence from the earned income tax credit. *Am. Econ. Rev.* 102 1927–1956. 10.1257/aer.102.5.1927

[B28] Darmody M. Smyth E. (2008). Full-time students? Term-time employment among higher education students in Ireland. *J. Educ. Work* 21 349–362. 10.1080/13639080802361091

[B29] Deng W. Li X. Wu H. Xu G. (2020). Student leadership and academic performance. *China Econ. Rev.* 60:101389 10.1016/j.chieco.2019.101389

[B30] Denning J. T. (2017). Born under a lucky star financial aid, college completion, labor supply, and credit constraints. *J. Hum. Resour.* 54 760–784. 10.17848/wp17-267

[B31] Dovey T. Ludgate A. Tutak J. (2017). *Success by Design. Improving outcomes in American Higher Education. A Deloitte Center for Higher Education Excellence Series On Student Success.* New York, NY: Deloitte University Press.

[B32] Dundes L. Marx J. (2006). Balancing work and academics in college: Why do students working 10 to 19 hours per week excel? *J. Coll. Stud. Retent.* 8 107–120. 10.2190/7UCU-8F9M-94QG-5WWQ 22612255

[B33] Dziuban C. Moskal P. (2011a). A course is a course is a course: Factor invariance in student evaluation of online, blended and face-to-face learning environments. *Int. High. Educ.* 14 236–241. 10.1016/j.iheduc.2011.05.003

[B34] Dziuban C. Moskal P. (2011b). *Technology-enhanced learning: Opportunities and challenges Presented at the Faculty Conference on Teaching Effectiveness.* Miami, FL: University of Central Florida.

[B35] Edwards A. Baker S. (2014). Factor Caribbean Overseas Students Perceive Influence their Academic Self- efficacy. *J. Int. Stud.* 4 48–59.

[B36] Fazio M. (2004). *Incidence of Worked Hours on the Academic Performance of Argentine University Students [Incidencia de las horas trabajadas en el rendimiento académico de estudiantes universitarios argentinos].* Master Thesis, Universidad Nacional de La Plata, Argentina.

[B37] Francis M. K. Wormington S. V. Hulleman C. (2019). The costs of online learning: Examining differences in motivation and academic outcomes in online and face-to-face community college developmental mathematics courses. *Front. Psychol.* 10:2054. 10.3389/fpsyg.2019.02054 31551886 PMC6746985

[B38] Fuse A. (2018). Needs of students seeking careers in communication sciences and disorders and barriers to their success. *J. Commun. Disord.* 72 40–53. 10.1016/j.jcomdis.2018.02.003 29471177

[B39] Gamallo G. Y. Nuñez P. (2013). La aventura del héroe: proyectos migratorios de los estudiantes universitarios de Río Negro. *Trabajo Soc.* 20 71–98.

[B40] Gottardi M. D. (2015). A autonomia na aprendizagem em educação a distância: competência a ser desenvolvida pelo aluno. *Rev. Bras. Aprend. Aberta Distância* 14 107–120. 10.17143/rbaad.v14i0.268

[B41] Graziosi G. Sneyers E. Agasisti T. De Witte K. (2020). Can grants affect student performance? Evidence from five Italian universities. *J. High. Educ. Policy Manag.* 42, 1–25. 10.1080/1360080X.2020.1737343

[B42] Greenberger E. Steinberg L. D. (1986). *When Teenagers Work? The Psychological and Social Costs of Adolescent Employment.* New York: Basic Books.

[B43] Grilli L. Rampichini C. (2009). “Multilevel models for the evaluation of educational institutions: a review,” in *Statistical Methods for the Evaluation of Educational Services and Quality Of Products*, eds Monari P. Bini M. Piccolo D. Salmaso L., (Heidelberg: Springer Science & Bussines Media), 61–80. 10.1007/978-3-7908-2385-1_5

[B44] Hamilton L. T. (2013). More is more or more is less? Parental financial investments during college. *Am. Sociol. Rev.* 78 70–95. 10.1177/0003122412472680

[B45] Hardin C. J. (2008). Adult students in higher education: a portrait of transitions. *New Direct. High. Educ.* 144 49–57. 10.1002/he.325

[B46] Hossler D. Ziskin M. Gross J. P. (2009). Getting serious about institutional performance in student retention: Research-based lessons on effective policies and practices. *About Campus.* 13 2–11. 10.1002/abc.271

[B47] Instituto Colombiano para la Evaluación de la Educación – ICFES (2019). *Estructura general del examen Icfes Saber Pro - Portal Icfes.* Bogotá: ICFES.

[B48] Jongbloed B. Lepori B. (2015). “The funding of research in higher education: Mixed models and mixed results,” in *The Palgrave International Handbook of Higher Education Policy and Governance*, eds Huisman J. de Boer H. Dill D. Souto-Otero M., (London: Palgrave Macmillan), 439–462. 10.1007/978-1-137-45617-5_24

[B49] Kirmizi O. (2013). Investigating self-regulated learning habits of distance education students. *J. History Cult. Art Res.* 2 161–174. 10.7596/taksad.v2i2.246

[B50] Leaño A. Y. Jaramillo D. G. (2018). La autonomía del aprendizaje: el caso del programa Tecnología Empresarial en el municipio de San Alberto. *Cesar. Rev. Docencia Univ.* 19 19–29.

[B51] Martin J. Sugarman J. H. Hickinbottom S. (2010). *Persons: Understanding Psychological Selfhood and Agency.* New York, NY: Springer Science & Business Media.

[B52] McGoldrick M. García N. A. Carter B. A. (2016). *The Expanding Family Life Cycle: Individual, Family, and Social Perspectives*, 5th Edn New Jersey: Pearson.

[B53] Melguizo T. Sanchez F. Velasco T. (2016). Credit for low-income students and access to and academic performance in higher education in Colombia: A regression discontinuity approach. *World Dev.* 80 61–77. 10.1016/j.worlddev.2015.11.018

[B54] Mertens D. M. (2014). *Research and Evaluation in Education and Psychology: Integrating Diversity with Quantitative, Qualitative, and Mixed Methods*, 4th Edn Thousand Oaks, CA: Sage publications.

[B55] Mostrom A. M. Blumberg P. (2012). Does learning-centered teaching promote grade improvement? *Innovat. High. Educ.* 37 397–405. 10.1007/s10755-012-9216-1

[B56] Muñoz Montes M. M. Marín Catalán R. (2018). Programa de inclusión en Educación Superior: experiencias de estudiantes en la Facultad de Medicina. *Pensamiento Educ. Rev. Invest. Educ. Latinoamer.* 55 1–15. 10.7764/pel.55.1.2018.4

[B57] Murillo Torrecilla F. J. (2008). Los modelos multinivel como herramienta para la investigación educativa. *Magis. Rev. Int. Invest. Educ.* 1 45–62.

[B58] Nguyen T. D. Kramer J. W. Evans B. J. (2019). The effects of grant aid on student persistence and degree attainment: a systematic review and meta-analysis of the causal evidence. *Rev. Educ. Res.* 89 831–874. 10.3102/0034654319877156

[B59] Padgett R. D. Grady D. L. (2009). “Student development and personal growth in employment,” in *Enhancing Student Learning Through College Employment*, ed. Perozzi B., (Bloomington, IN: Association of College Unions International), 31–43.

[B60] Paul J. Jefferson F. (2019). A comparative analysis of student performance in an online versus face-to-face environmental science course from 2009-2016. *Front. Computer Sci.* 1:7 10.3389/fcomp.2019.00007

[B61] Rafidah K. Azizah A. Norzaidi M. Chonh S. Salwani M. Noraini I. (2009). Stress and Academic Performance: Empirical Evidence from University Students. *Acad. Educ. Leadersh. J.* 13 37–51.

[B62] Rangel M. A. Vázquez Flores F. Ruiz Vargas N. Juárez Nilo S. Hernández Segura G. Gallegos Torres R. (2019). Calidad de vida emocional en estudiantes foráneos de tres licenciaturas de una universidad pública del Estado de Querétaro, México. *Rev. Horizonte Enfermería* 30 16–26. 10.7764/Horiz_Enferm.30.1.16-26

[B63] Roddy C. Amiet D. L. Chung J. Holt C. Shaw L. McKenzie S. (2017). Applying best practice online learning, teaching, and support to intensive online environments: an integrative review. *Front. Educ.* 2:59 10.3389/feduc.2017.00059

[B64] Roksa J. Kinsley P. (2019). The Role of Family Support in Facilitating Academic Success of Low-Income Students. *Res. High. Educ.* 60 415–436. 10.1007/s11162-018-9525-z

[B65] Ryan R. M. Deci E. L. (2002). ““Overview of self-determination theory: An organismic dialectic perspective”,” in *Handbook of Self-Determination Research*, eds Decy E. Ryan R. M., (Rochester, NY: University of Rochester Press), 3–33.

[B66] Ryan R. M. Deci E. L. (2017). *Self-Determination Theory: Basic Psychological Needs in Motivation, Development and Wellness.* New York, NY: The Guilford Press.

[B67] Saavedra J. E. (2009). *The Learning and Early Labor Market Effects of College Quality: A Regression Discontinuity Analysis.* Bogotá: Universidad de los Andes, School of Government.

[B68] Salisbury M. H. Pascarella E. T. Padgett R. D. Blaich C. (2012). The effects of work on leadership development among first-year college students. *J. Coll. Stud. Dev*. 53 300–324. 10.1353/csd.2012.0021

[B69] Sauerwein M. (2017). *Qualität in Bildungssettings der Ganztagsschule. Über Unterrichtsforschung und Sozialpädagogik.* Switzerland: Beltz Juventa.

[B70] Shim S. Barber B. L. Card N. A. Xiao J. J. Serido J. (2010). Financial socialization of first-year college students: The roles of parents, work, and education. *J. Youth Adolesc.* 39 1457–1470. 10.1007/s10964-009-9432-x 20938727

[B71] Shim S. Xiao J. J. Barber B. L. Lyons A. C. (2009). Pathways to life success: a conceptual model of financial well-being for young adults. *J. Appl. Dev. Psychol.* 30, 708–723. 10.1016/j.appdev.2009.02.003

[B72] Torcomian C. G. (2016). Experiencias universitarias en estudiantes migrantes. *Rev. Invest. Psicol.* 19 49–68. 10.15381/rinvp.v19i2.12889

[B73] Toro J. R. (2004). La autonomía, el propósito de la educación. *Rev. Estudios Soc*. 19 119–124.

[B74] Torres G. Y. Parra L. S. (2019). Consejería psicológica virtual en la universidad colombiana: más allá del rendimiento académico. *Rev. Invest. And.* 21 113–124. 10.33132/01248146.994

[B75] Vera P. B. (2014). “La autonomía del estudiante dentro del espacio social y académico en la Universidad Veracruzana”Las dimensiones y regulaciones disciplinarias. *Ciencia Huasteca Bol. Científico Escuela Super. Huejutla* 2:4 10.29057/esh.v2i4.1073

[B76] Vicencio J. R. Banaag A. G. (2019). Balancing Work and Studies: The Omani Students’perspective. *Eur. J. Educ. Stud.* 6 328–340.

[B77] Wang H. Kong M. Shan W. Vong S. K. (2010). The effects of doing part-time jobs on college student academic performance and social life in a Chinese society. *J. Educ. Work* 23 79–94. 10.1080/13639080903418402

[B78] Watson S. J. Barber B. L. (2017). University attendance moderates the link between financial norms and healthy financial behavior for australian young adults. *J. Fam. Econ. Issues* 38 238–248. 10.1007/s10834-016-9505-4

[B79] Wenz M. Yu W. (2010). Term-time Employment and The Academic Performance of Undergraduates. *J. Educ. Finance* 35 359–374.

[B80] Xiao J. (2018). On the margins or at the center? Distance education in higher education. *J. Distance Educ.* 39 259–274. 10.1080/01587919.2018.1429213

[B81] Yu S. Levesque-Bristol C. (2018). Are students in some college majors more self-determined in their studies than others? *Motiv. Emot.* 42 831–851. 10.1007/s11031-018-9711-5

[B82] Yu S. Levesque-Bristol C. (2020). A cross-classified path analysis of the self-determination theory model on the situational, individual and classroom levels in college education. *Contemp. Educ. Psychol.* 61:101857 10.1016/j.cedpsych.2020.101857

[B83] Yu S. Levesque-Bristol C. Maeda Y. (2018). General need for autonomy and subjective well-being: A meta-analysis of studies in the US and East Asia. *J. Happiness Stud.* 19 1863–1882. 10.1007/s10902-017-9898-2

[B84] Yuan J. Kim C. (2018). The effects of autonomy support on student engagement in peer assessment. *Educ. Technol. Res. Dev.* 66 25–52. 10.1007/s11423-017-9538-x

[B85] Zhu W. Yoo J. Curry J. C. Chu H. W. Craig B. Chu H. (2019). “The results and impact of awarding S-STEM scholarships to current students,” *Paper presented at 126TH Annual Conference and Exposition of American Society for Engineering Education*, Florida.

[B86] Zimmerman B. J. (2002). Becoming a self-regulated learner: an overview. *Theor. Pract.* 41 64–70. 10.1207/s15430421tip4102_2

